# Discovery of Evolutionary Loss of the Ubiquitin-like Autophagy-Related ATG12 System in a Lineage of Apicomplexa

**DOI:** 10.3390/cells14020121

**Published:** 2025-01-15

**Authors:** Xiaoxia X. Lin, Yun D. Bai, Sichang T. Wang, Akira Nozawa, Tatsuya Sawasaki, Tatsunori Masatani, Kenji Hikosaka, Masahito Asada, Hirokazu Sakamoto

**Affiliations:** 1 Department of Infection and Host Defense, Graduate School of Medicine, Chiba University, Chiba 263-8522, Japan; lxx870611@yahoo.co.jp (X.X.L.); baiyun426@yeah.net (Y.D.B.); 23fm0104@student.gs.chiba-u.jp (S.T.W.); hikosaka@chiba-u.jp (K.H.); 2 Proteo-Science Center (PROS), Ehime University, Matsuyama 790-0825, Japan; nozawa.akira.my@ehime-u.ac.jp (A.N.); sawasaki.tatsuya.mf@ehime-u.ac.jp (T.S.); 3 Laboratory of Zoonotic Diseases, Faculty of Applied Biological Sciences, Gifu University, Gifu 501-1193, Japan; masatani.tatsunori.j9@f.gifu-u.ac.jp; 4 Joint Graduate School of Veterinary Sciences, Gifu University, Gifu 501-1193, Japan; 5 Division of Animal Medical Science, Center for One Medicine Innovative Translational Research (COMIT), Institute for Advanced Study, Gifu University, Gifu 501-1193, Japan; 6 National Research Center for Protozoan Diseases, Obihiro University of Agriculture and Veterinary Medicine, Hokkaido 080-0834, Japan; masada@obihiro.ac.jp

**Keywords:** autophagy, ATG12 system, Apicomplexa, piroplasmida

## Abstract

The autophagy-related ubiquitin-like conjugation systems, the ATG8 and ATG12 systems, are universally conserved in eukaryotes. However, the covalent bond in the ATG12 system has recently been shown to be evolutionarily lost in Apicomplexa. Here, we show that all genes associated with the ATG12 system are absent in piroplasmida, a lineage within Apicomplexa. Comparative genomics of ATGs further shows that piroplasm ATG3 has lost the region necessary for ATG12 binding. However, our in vitro functional analysis using recombinant proteins demonstrated that ATG3 retained the ability to interact with ATG8 in *Babesia bovis*, a model species in piroplasmida. These findings provide evidence that the ATG8 system is functional, while the ATG12 system is completely lost in the common ancestor of piroplasmida and highlight the evolutionary flexibility of the ATG12 system in Apicomplexa.

## 1. Introduction

**Figure 1:**
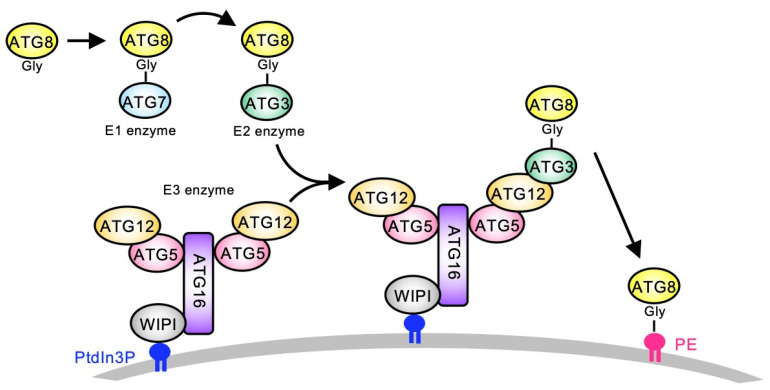
The ATG12-ATG5-ATG16 complex recruits ATG8-ATG3 to the membrane surface. A schematic diagram of the process of the ATG12 and ATG8 systems. A UBL protein, ATG8, conjugates with phosphatylethanolamine (PE) via the E1-like ATG7, E2-like ATG3, and E3-like ATG12-ATG5-ATG16 complex. Another UBL protein, ATG12, conjugates with ATG5 via the E1-like ATG7 and E2-like ATG10. The ATG12–ATG5 conjugate forms a dimer mediated by ATG16, called the ATG12-ATG5-ATG16 complex. The complex localizes on the membrane mediated by WD-repeat protein interacting with phosphoinositides (WIPI), which recognizes phosphatidylinositol 3-phosphate (PtdIn3P). The membrane-localized ATG12-ATG5-ATG16 complex recognizes the ATG8-ATG3 on the membrane surface.

Macroautophagy, usually simply called ’autophagy’, is an intercellular degradation system that can degrade large cytosolic components, including organelles. Autophagy is mediated by double-membrane structures called autophagosomes [1]. This process is mediated by autophagy-related (ATG) proteins, which are largely conserved among eukaryotes [2]. Among these, two ubiquitin-like (UBL) conjugation systems—the ATG12 and ATG8 systems—play pivotal roles in autophagosome formation [3,4]. ATG12 and ATG8 are UBL proteins (Figure 1). ATG12 conjugates ATG5 via the E1-like enzyme ATG7 and the E2-like enzyme ATG10. Then, the ATG12–ATG5 conjugate forms a dimer via ATG16, called the ATG12-ATG5-ATG16 complex. Another UBL protein, ATG8, conjugates phosphatidylethanolamine (PE) via the E1-like enzyme ATG7, the E2-like enzyme ATG3, and the E3-like enzyme ATG12-ATG5-ATG16 complex. The E3-like complex localizes on the membrane via phosphatidylinositol 3-phosphate (PtdIn3P) and WD-repeat protein interacting with phosphoinositides (or Atg18). Then, ATG12 recognizes the ATG8-ATG3 complex and conjugates ATG8 to PE on the membrane. Finally, the ATG8–PE conjugate localizes on the autophagosome membrane and plays a critical role in autophagosome formation.

Although these reactions are essential for the autophagy process, recent studies have shown that the UBL-like conjugation reactions of the ATG12 system are evolutionarily lost in certain eukaryotes [5,6,7]. For example, Apicomplexa, including malaria parasites and *Toxoplasma gondii*, and the yeast *Komagataella phaffii* independently evolved non-covalent ATG12-ATG5 complexes in place of the covalent conjugation [5]. They lose ATG10, but their ATG12 can bind to ATG5 without the UBL conjugation reaction. Moreover, such losses appear to have occurred independently at least 16 times among representative 94 eukaryotes from every supergroup [7].

Apicomplexan parasites are attractive research targets for understanding the functional and molecular mechanistic diversity of ATG proteins [8,9]. They differ from other eukaryotes not only in the loss of the covalent conjugation of ATG12 but also in the localization and function of ATG8. Apicomplexan ATG8 localizes on the outermost membrane of the apicoplast, a non-photosynthetic plastid-derived organelle [10,11,12]. This unique localization of ATG8 is essential for maintaining apicoplast morphology and inheritance, rather than autophagic degradation [11,13]. Although localized to an organelle distinct from the autophagosome, the localization of ATG8 to the apicoplast is regulated by the ATG12-ATG5-ATG16 complex, as in model organisms [14]. This suggests that localization control of ATG8 is conserved beyond its functional divergence and eukaryotic supergroups.

In this study, we found that the previously established findings regarding the ATG12 system do not apply to piroplasmida, a subgroup of Apicomplexa. Our homology search showed that the genes encoding the ATG12-ATG5-ATG16 complex have been lost in the common ancestor of piroplasms. Additionally, sequence analysis of piroplasm ATG3 and its predicted interaction motifs strongly supported the loss of ATG12. From these results, we conclude that piroplasms have no ATG12 system. This study highlights the evolutionary flexibility of the ATG12 system within the apicomplexan lineages.

## 2. Materials and Methods

### 2.1. Homology Search

We searched, using BLASTp and tBLASTn, for the ATG homologs related to the ATG8 and ATG12 conjugation systems in the apicomplexan parasites using PlasmoDB (https://plasmodb.org/plasmo/app; accessed on 10 January 2025), PiroplasmaDB (https://piroplasmadb.org/piro/app; accessed on 10 January 2025), and ToxoDB (https://toxodb.org/toxo/app; accessed on 10 January 2025). We focused on haemosporidia, piroplasmida, and coccidia since they possess the apicoplast. We selected four species of *P. falciparum*, *P. vivax*, *P. yoelii*, and *P. gallinaceum* from different phylogenetic lines as representatives of the genus *Plasmodium*. ATGs in *P. falciparum* were used as queries for *Plasmodium* and piroplasms. ATGs in *T. gondii* were used as queries for coccidia. Full-length amino acid sequences of the ATGs were used for each search. Note, however, that in the search for ATG12 homologs for coccidia, searches were also conducted in the conserved ATG12 region of TgATG12 (572-681 aa) [5].

### 2.2. Search for the AIM8 and AIM12 Motifs in ATG3 Homologs

We predicted the ATG8-interacting motif (AIM8) and ATG12-interacting motif (AIM12) in ATG3 homologs using AlphaFold Server (https://alphafoldserver.com, accessed on 10 January 2025) [15]. The full-length amino acid sequences were used for the prediction. For the prediction of AIM8 in ATG3 homologs, the ATG8–ATG3 interaction in *P. falciparum* was used as a positive control because the crystal structure of the interaction has already been experimentally solved [16]. For the prediction of AIM12 in ATG3 homologs, the ATG12–ATG3 interaction in a plant (*Arabidopsis thaliana*) was used as a positive control because the crystal structure of the interaction has already been experimentally solved [17].

### 2.3. Cell Culture and cDNA Preparation of Babesia bovis

*Babesia bovis* (Texas strain) cells were cultured in GIT medium (Fujifilm Wako Pure Chemical, Osaka, Japan) with purified bovine red blood cells (Japan BioSerum, Hiroshima, Japan) at 10% hematocrit. The cells were incubated in a microaerophilic stationary-phase culture system. Total RNA was extracted from parasite pellets using TRIzol LS reagent (ThermoFisher Scientific, Waltham, MA, USA) according to the manual. cDNA was synthesized from the total RNA using the PrimeScript II 1st Strand cDNA Synthesis Kit (Takara Bio, Shiga, Japan) according to the manual.

### 2.4. Plasmids

The coding sequences for ATG3 and ATG8 in *B. bovis* (Bb) and *P. falciparum* (Pf) were amplified by PCR from the cDNA and codon-optimized synthetic DNA, respectively. Used primers are listed in Table S1. BbATG3 and BbATG8 genes were amplified by 2-step PCR and cloned into a pMRX-IPU-3xFLAG vector. Then, they were subcloned into pEU vectors (CellFree Sciences, Matsuyama, Japan) for wheat germ cell-free expression. Genes for ATG3 and ATG8 were inserted into the NotI-cut pEU-E01-bls-MCS(N2) and NotI-cut pEU-E01-FLAG-MCS(N2) vectors, respectively.

For mutants of ATG8-interacting motif (AIM8) in ATG3 homologs, a PrimeSTAR Mutagenesis Basal Kit (Takara Bio, Shiga, Japan) was used with the mutagenesis primers (Table S1). The plasmids produced in this study and their names are listed in Table S2.

### 2.5. Wheat Germ Cell-Free Protein Synthesis

ATG3 and ATG8 recombinant proteins were synthesized by the wheat germ cell-free synthesis system [18]. The WEPRO1240 expression kit (CellFree Sciences, Matsuyama, Japan) was used according to the manual. BirA biotin ligase was added into the translation mixture for biotinylation of ATG3 proteins. After the translation reaction, the mixtures were aliquoted and stored at −80 °C until use.

For biotin-ATG3 proteins, we used a part of the translation mixtures (total fraction) for the preparation of a supernatant fraction. The total fractions were centrifuged at 15,000× *g* for 20 min at 4 °C, and the supernatant was collected. We used the total and supernatant fractions for western blotting.

### 2.6. Western Blot

The synthesized proteins were subjected to SDS-PAGE, with the proper concentration of polyacrylamide gel. After electrophoresis, the proteins in the gel were transferred to a PVDF membrane (Merk Millipore, Burlington, MO, USA). The transferred membrane was reacted with blocking buffer (5% skim milk/TBST (150 mM NaCl, 20 mM Tris-HCl (pH 7.5), 0.05% Tween-20) for 1 h at room temperature. Then, the membrane was treated with HRP-conjugated anti-biotin antibody (Cell Signaling Technology, Danvers, MA, USA, #7075, 1:5000) or anti-FLAG antibody (Sigma Aldrich, Burlington, MO, USA, A8592, 1:5000) for 1 h at room temperature. After washing the membrane with TBST for 10 min three times, the membrane was treated with Immobilon Western Chemiluminescent HRP Substrate (Merck Millipore, Burlington, MO, USA) according to the manufacturer’s instructions. The signals were visualized with an Image Quant LAS 4000 mini (GE Healthcare, Buckinghamshire, UK). Acquired images were processed using ImageJ software v1.54g (National Institutes of Health, Rockville, MD, USA).

### 2.7. AlphaScreen

For detection of the interaction between FLAG-ATG8 and biotin-ATG3, we performed the AlphaScreen system (Revvity Japan, Yokohama, Japan). The total fraction of the translation mixtures was used in the system. Firstly, 15 µL of a reaction mixture containing AlphaScreen buffer (100 mM Tris-HCl (pH 8.0), 0.1% Tween-20, 1 mg/mL bovine serum albumin), 0.5 µL of biotin-ATG3, and 0.125 µL of FLAG-ATG8 proteins was added to a 384-well Optiplate (Revvity, Japan, Yokohama, Japan). After incubation at 26 °C for 1 h, 10 µL of a detection mixture containing AlphaScreen buffer, 0.1 µL of streptavidin-coated donor beads (Revvity Japan, Yokohama, Japan), 0.1 µL of protein A-coated acceptor beads (Revvity Japan, Yokohama, Japan), and 25 ng of anti-FLAG M2 antibody (Sigma Aldrich, Burlington, MO, USA) was added to each well. After incubation at 26 °C for 1 h, luminescence signals were detected using an Envision plate reader (Revvity Japan, Yokohama, Japan). The experiment was repeated three times in triplicate.

## 3. Results

### 3.1. The ATG8 Conjugation System Is Conserved in Haemosporidia, Piroplasmida, and Coccidia

**Figure 2:**
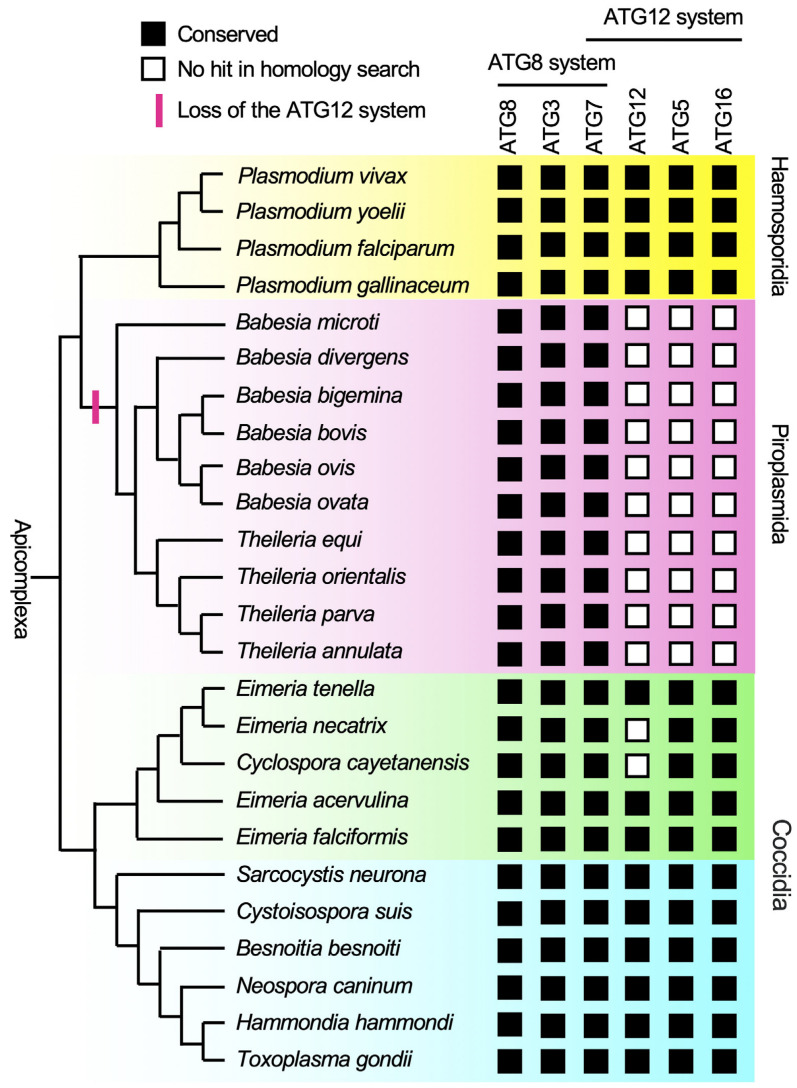
The genes for the ATG12 system have been lost in piroplasmida. A simplified phylogenic tree of Apicomplexa and conservation of genes for the ATG8 and ATG12 systems. Haemosporidia, piroplasmida, and coccidia are shown. Conserved and non-conserved genes are shown in black and white boxes, respectively.

We first searched for ATG8 homologs and focused our analysis on species where ATG8 was detected (Figure 2, Table S3). Sequence alignment of the identified apicomplexan ATG8 homologs revealed a high conservation of amino acid sequences within each genus (Figure S1). Previously, it was thought that apicomplexan ATG8 proteins, based on sequences from *Plasmodium* and *Toxoplasma*, had their C-terminal glycine (required for conjugation to PE) exposed. Interestingly, however, ATG8 in *Babesia microti* (BMR1_03g03150) and *Sarcocystis neurona* (SN3_00202075) contained an extended sequence downstream of the C-terminal glycine. Additionally, ATG8 in *Sarcocystis neurona* and *Cystoisospora suis* (CSUI_000409) have an extension sequence at their N-terminal region. Based on the phylogenic relationships of the parasites, these extension sequences are considered to have been acquired independently from each other.

We then searched for other components of the ATG8 conjugation system (ATG7 and ATG3) in the species where ATG8 was identified. These factors were detected in all species (Figure 2, Table S3). However, the BLASTp search did not identify ATG3 in *Babesia ovata*, which was identified by the tBLASTn search.

Taken together, these data suggest that apicomplexan parasites generally conserve the ATG8 conjugation system. The high degree of sequence conservation in ATG8 underscores the strong evolutionary pressure to maintain its function, indicating its universal and critical role in Apicomplexa.

### 3.2. Complete Loss of the ATG12 System in Piroplasmida

To elucidate the diversity of the ATG12 sequences in Apicomplexa, we searched for ATGs for the ATG12 system (ATG5, ATG12, and ATG16) using PlasmoDB, ToxoDB, and PiroplasmaDB. In our homology search, all of the ATGs involved in the ATG12 system were not found in piroplasms (*Babesia* and *Theileria* genera) (Figure 2, Table S3). The results suggest that the ATG12 system has been lost in the common ancestor of piroplasms.

Our BLASTp and tBLASTn search did not identify any ATG12 homologs in *Eimeria necatrix* and *Cyclospora cayetanensis*, even when using the ubiquitin fold region of TgATG12 (572-681 aa) [5] as a query. However, the absence of ATG12 remains a possibility due to dataset limitations rather than a true absence, as ATG5 was identified from these species with high amino acid sequence similarity to the ATG5 homologs in the *Eimeria* genus (Figure S2).

In contrast, our findings strongly suggest that the absence of ATG factors associated with the ATG12 system in all piroplasm species reflects an evolutionary loss in their common ancestor, rather than a false negative due to database limitations.

### 3.3. Piroplasm ATG8 Homologs Conserve the Ubiquitin Fold Structure but Possess Unique N-Terminal Sequences

**Figure 3:**
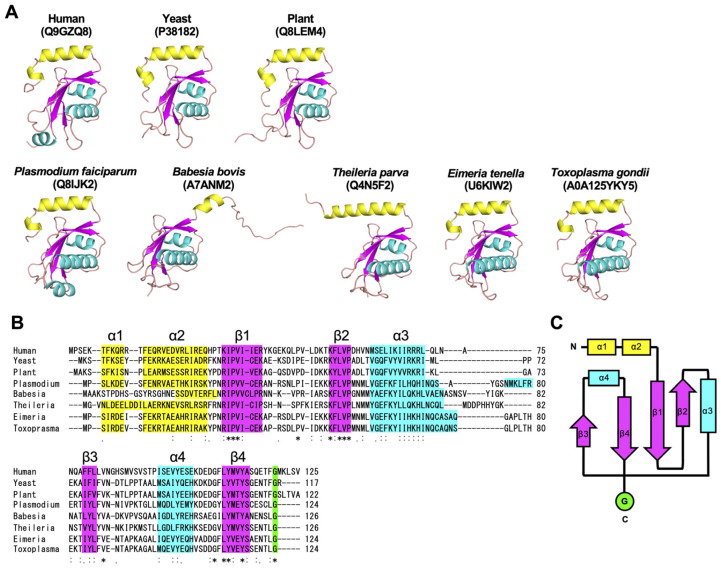
Structural conservation of ATG8 homologs in Apicomplexa. (**A**) Three-dimensional structures of full-length ATG8 homologs among model organisms and apicomplexans predicted by AlphaFold. The UniProt numbers that can be used for searching in the AlphaFold Protein Structure Database (https://alphafold.ebi.ac.uk/, accessed on 10 January 2025) are shown. (**B**) Amino acid alignment of ATG8 homologs among model organisms and apicomplexans. Plasmodium: *Plasmodium falciparum*, Babesia: *Babesia bovis*, Theileria: *Theileria parva*, Eimeria: *Eimeria tenella*. The color corresponds with (**A**). (**C**) A schematic diagram of 3D structures conserved in ATG8. The color of each box corresponds with (**A**). (**A**–**C**) Yellow: ATG8-specific N-terminal α helix, cyan: α helix, magenta: β sheet. Human: *H. sapiens*, yeast: *S. cerevisiae*, plant: *A. thaliana*. “*” indicates identical residues across all sequences “:” denotes residues with high chemical similarity and ≥70% similarity “.” represents residues with moderate similarity and 50–70% similarity.

In the event that the ATG12 lineage is lost in piroplasms, the concern is whether the conserved factors of the ATG8 system retain functionality or whether they have evolved into pseudogenes. Therefore, to infer the functionality of ATG8 in piroplasm species lacking the ATG12 system, we compared the sequences and AlphaFold-predicted structures of ATG8 from the type species of *Babesia* and *Theileria*, *Babesia bovis* (BbATG8) and *Theileria parva* (TpATG8) (Figure 3). As comparators, we used ATG8 homologs from model organisms (human, yeast, and plant) and other apicomplexans (*P. falciparum*, *T. gondii*, and *Eimeria tenella*). All the AlphaFold-predicted structures are based on predictions from full-length sequences. Previous structural studies have shown that ATG8 homologs conserve the N-terminal helix and the ubiquitin fold structure [19]. The predicted structures also exhibited these conserved features and accurately reproduced the crystal structure data (Figure 3A). The model confidence (the predicted local distance difference test (pLDDT)) of all predicted structures was quite high (Figure S3A). However, the pLDDT values for Apicomplexa-specific insertion sequences downstream of the α3 were somewhat lower. Both BbATG8 and TpATG8 were predicted to have a ubiquitin fold structure (Figure 3A), and their secondary structures perfectly matched those of typical ATG8 homologs (Figure 3B). These results strongly suggest that ATG8 may be functional in piroplasmida.

On the other hand, piroplasm ATG8 differed from other ATG8 homologs in the N-terminal helix structure; BbATG8 had a short helix length, and TpATG8 had only one helix (Figure 3A). Because of the low pLDDT values of these regions (Figure S3A), we cannot exclude the possibility that they have typical structures. Although the amino acidsequence of these N-terminal helices is highly conserved among model organisms, *Plasmodium*, and coccidia, the piroplasm ATG8 homologs have unique sequences (Figure S1).

### 3.4. Piroplasm ATG3 Conserves the E2-Core Structure, but Its Flexible Region Is Shorter

**Figure 4:**
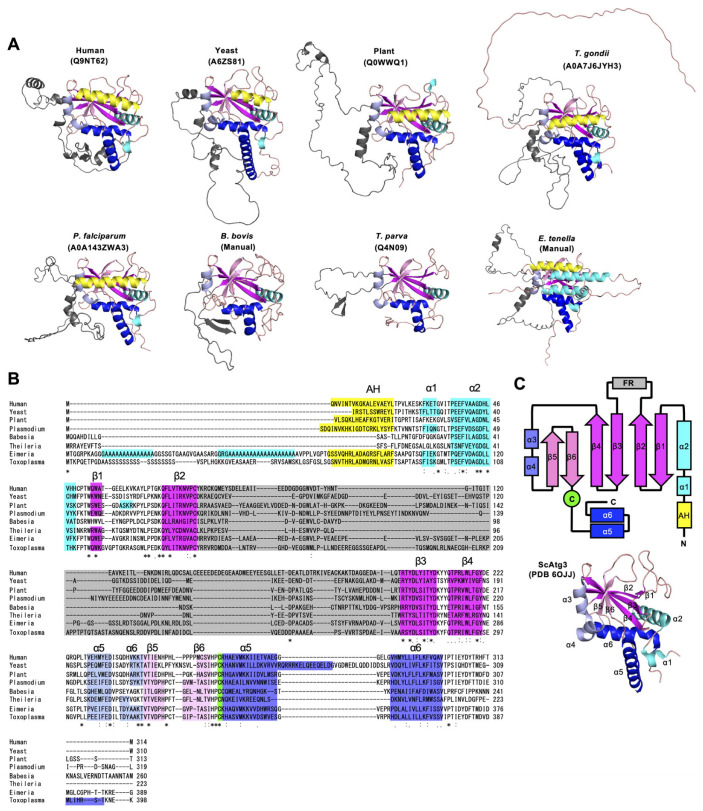
Structural conservation of ATG3 homologs. (**A**) Three-dimensional structures of full-length ATG3 homologs among model organisms and apicomplexans predicted by AlphaFold. The UniProt numbers that can be used for searching in the AlphaFold Protein Structure Database (https://alphafold.ebi.ac.uk/) are shown. Prediction of the ATG3 structures of *B. bovis* and *E. tenella* were performed manually using AlphaFold server. (**B**) Amino acid alignment of ATG3 homologs among model organisms and apicomplexans. Plasmodium: *Plasmodium falciparum*, Babesia: *Babesia bovis*, Theileria: *Theileria parva*, Eimeria: *Eimeria tenella*. The color corresponds with (**A**). (**C**) A schematic diagram of 3D structures conserved in ATG3 (upper) and the E2-core structure of yeast Atg3 (lower; PDB 6OJJ). The color of each box corresponds with (**A**). FR: flexible region. (**A**–**C**) Yellow: amphipathic α helix (AH), cyan or blue: α helix, magenta or pink: β sheet, gray: loop region. Human: *H. sapiens*, yeast: *S. cerevisiae*, plant: *A. thaliana*. “*” indicates identical residues across all sequences “:” denotes residues with high chemical similarity and ≥70% similarity “.” represents residues with moderate similarity and 50–70% similarity.

Next, we examined the structural conservation of piroplasm ATG3. The canonical ATG3 features an E2-core structure and intrinsically disordered regions (IDRs) [20,21] (Figure 4C). The full-length ATG3 homologs from model organisms predicted by AlphaFold also exhibited the E2-core structure, which closely resembled the crystal structure data (Figure 4A), with the pLDDT value exceeding 70 in this region (Figure S3B). Similarly, the E2-core structure was predicted with high pLDDT among apicomplexan ATG3 homologs (Figure 4A,B and Figure S3B), suggesting that ATG3 is structurally conserved in piroplasmida. Furthermore, the position of the catalytic cysteine, which interacts with the C-terminal glycine of ATG8, was also conserved. The conserved catalytic cysteine in ATG3 is critical for the ATG8 conjugation system, further suggesting that piroplasm ATG3 is likely functional, similar to its role in other organisms. On the other hand, however, the characteristic amphipathic helix at the N-terminus of ATG3 was not predicted in piroplasmida; also, the flexible region (FR) is shorter than other ATG3 homologs (Figure 4B).

**Figure 5:**
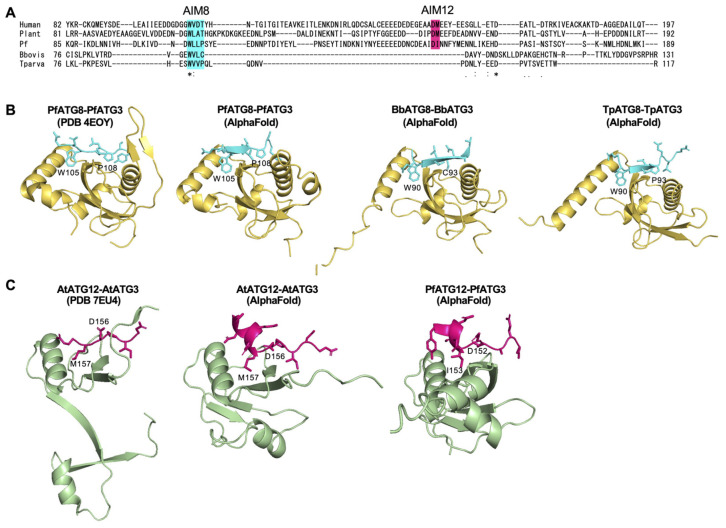
Alignment of the loop region of ATG3 homologs. (**A**) Amino acid alignment of the loop region in ATG3 homologs among model organisms and apicomplexans. AIM8 and AIM12 are highlighted in cyan and magenta, respectively. (**B**,**C**) Interaction regions of ATG8-ATG3 (**B**) and ATG12-ATG3 (**C**). Crystal structure data are shown on the leftmost panel, and AlphaFold predictions are shown on the right. Yellow: ATG8, cyan: AIM8, green: ATG12, magenta: AIM12. “*” indicates identical residues across all sequences “:” denotes residues with high chemical similarity and ≥70% similarity “.” represents residues with moderate similarity and 50–70% similarity.

The FR of ATG3 interacts with both ATG8 and ATG12 [22] (Figure 1), serving as a critical mediator for the ATG12 and ATG8 systems [23]. The FR contains specific amino acid motifs essential for binding, such as the ATG8-Interacting Motif (AIM, also referred to as LC3-Interacting Region, LIR) and the ATG12-Interacting Motif (AIM12) [17,20]. To avoid confusion between the AIM and AIM12, we hereafter refer to AIM as AIM8 in this study. Importantly, we noticed that the FR of the piroplasm ATG3 homologs is shorter than the typical ATG3 homologs (Figure 4B and Figure 5A). This indicates that piroplasm ATG3 may have lost its ability to bind ATG8 or ATG12, and examining whether AIM8 and AIM12 are conserved may allow us to evaluate the conservation and functionality of the ATG8 and ATG12 systems in piroplasmida.

### 3.5. ATG8 Interaction Motif (AIM8) in the Flexible Region of ATG3 Was Conserved Among ATG3 Homologs in Apicomplexa

To examine the presence of AIM8 in ATG3 homologs in piroplasmida, we assessed the conservation of AIM8 using 3D structural prediction methods. In *Plasmodium falciparum* (Pf), the AIM8 sequence in PfATG3 (WLLP) has been previously reported (PDB 4EOY) [16,24]. We validated the accuracy of AlphaFold in predicting the AIM8 sequence in PfATG3. Structural prediction of the PfATG8-PfATG3 complex using AlphaFold revealed that the predicted AIM8 sequence was consistent with those reported in earlier studies (Figure 5A,B), with the pLDDT value exceeding 90 (Figure S4A). This highlights the utility of AlphaFold in accurately predicting AIM8 in ATG3. Subsequently, we employed AlphaFold to predict AIM8 in *Babesia bovis* (Bb) and *Theileria parva* (Tp). The sequences WVLC and WVVP were identified with very high pLDDT as AIM8 in BbATG3 and TpATG3, respectively (Figure 5B and Figure S4A). Moreover, the positions of these AIM8 motifs were found to be conserved across other ATG3 homologs (Figure 5A). These results strongly suggest that AIM8 is conserved in piroplasm ATG3s, supporting the notion that ATG3 retains the ability to interact with ATG8.

### 3.6. ATG12 Interaction Motif (AIM12) in ATG3 Was Missing in Piroplasm ATG3

Next, we investigated whether AIM12 is conserved in piroplasmida. While AIM12 has not been identified in Apicomplexa, the Asp-Met sequence is conserved as AIM12 in humans and plants [17,25]. To evaluate the predictability of AIM12 using AlphaFold, we examined the ATG12–ATG3 interaction in the plant *Arabidopsis thaliana*. The sequences and position of predicted AIM12 matched those previously reported [17] (Figure 5C), with the pLDDT value exceeding 70 (Figure S4B). This demonstrates the utility of AlphaFold in predicting AIM12. We then predicted the PfATG12-PfATG3 complex structure and found that PfATG3 also contained an AIM12-like motif at a position analogous to that in humans and plants (Figure 5A,C), with the pLDDT value exceeding 70 (Figure S4B). The predicted AIM12 in PfATG3 featured an isoleucine residue binding to the hydrophobic pocket of PfATG12, replacing the methionine residue found in humans and plants. This substitution is likely functional, as both residues possess hydrophobic side chains, facilitating interaction with the hydrophobic pocket of ATG12. Importantly, no sequence corresponding to AIM12 was found in piroplasm ATG3 (Figure 5A). This finding strongly suggests that piroplasm ATG3 lacks AIM12, correlating with the absence of ATG12 system genes in them.

### 3.7. BbATG3 Binds to BbATG8 Depending on Its AIM8

**Figure 6:**
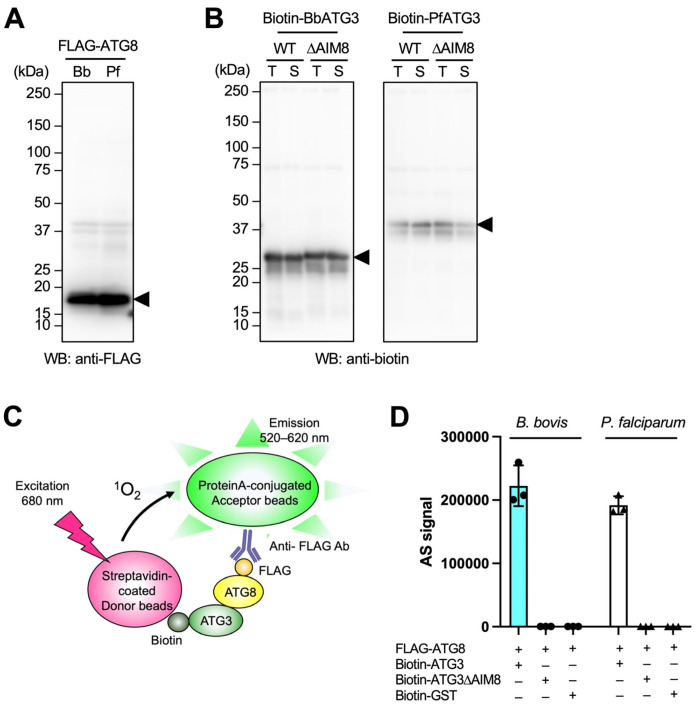
BbATG3 binds to BbATG8 depending on the AIM8. Preparation of FLAG-ATG8 (**A**) and biotin-ATG3 (**B**) recombinant proteins by wheat germ cell-free system and confirmation of synthesis by western blotting (arrowhead). For ATG3 (**B**), the total translation reaction solution (T) and the supernatant after centrifugation (S) were used to confirm the solubility of the mutants. (**C**) Schematic of the binding detection of FLAG-ATG8 and biotin-ATG3 by AlphaScreen. The method uses two types of dedicated detection beads, a donor and an acceptor bead. A 680 nm light excites the donor bead, which generates singlet oxygen, which is received by the acceptor bead, generating 520–620 nm fluorescence. Since the fluorescence intensity depends on the distance between these two types of beads, the interaction between the two target molecules can be evaluated. The donor bead is coated with streptavidin, which recognizes biotin-ATG3. The other acceptor bead is conjugated with ProteinA, which recognizes the anti-FLAG antibody, and the acceptor bead binds FLAG-ATG8 via the anti-FLAG antibody. (**D**) Results of binding tests with AlphaScreen for FLAG-ATG8 and biotin-ATG3 in *Babesia bovis* and *Plasmodium falciparum*. The vertical axis shows the AlphaScreen signal value (AS signal) and the horizontal axis shows the combination of the proteins. Biotin-GST was used as a negative control for Biotin-ATG3. Experiments were performed in triplicate at three independent times, and the mean of the triplicate for each experiment is indicated by dots. Error bars indicate SD.

To determine the functionality of the ATG8 conjugation system in piroplasmida, we examined the binding ability between ATG3 and ATG8 in *Babesia bovis* (Bb) as a representative of piroplasms. And PfATG8 and PfATG3 were used as positive controls. FLAG-ATG8 and biotin-ATG3 recombinant proteins were expressed in the wheat germ cell-free system and confirmed by western blot (Figure 6A,B). The interaction was evaluated by AlphaScreen (Figure 6C). When FLAG-BbATG8 and biotin-BbATG3 were incubated together, binding signals of comparable intensity to those of FLAG-PfATG8 and biotin-PfATG3 were observed (Figure 6D). No signals were detected with biotin-GST as a negative control. This demonstrates that a stable binding interaction exists between ATG8 and ATG3 in *B. bovis*.

Subsequently, we investigated the role of AIM8 in BbATG3 for the interaction. AIM8 in BbATG3 was mutated from WVLC to AVLA (BbATG3ΔAIM8), and in PfATG3, the mutation was made from WLLP to ALLA (PfATG3ΔAIM8). Mutations in IDRs, such as the FR of ATG3, may influence protein solubility. To assess the effect of the AIM8 deletion on solubility, we conducted a western blot analysis of total and supernatant fractions. The results showed that signals in the supernatant fractions were of comparable intensity to those in the total fractions, demonstrating that the AIM8 deletion does not impair the solubility of ATG3 proteins (Figure 6B). The AIM8-deleted biotin-ATG3s showed no detectable binding to ATG8 in either *B. bovis* or *P. falciparum* (Figure 6D). These results indicate that the interaction between ATG8 and ATG3 in *B. bovis* is comparable to that in *P. falciparum* and is dependent on the AIM8.

## 4. Discussion

Since the ATG8 and ATG12 conjugation systems are essential for autophagy in model organisms, the ATGs involving these systems are thought to be universally conserved across eukaryotes. However, previous studies have revealed the loss of ATG10, the E2-like enzyme in the ATG12 conjugation system, in several eukaryotic lineages, highlighting the diversity within the ATG12 system [5,6,7]. In this study, we present two key findings: our homology search did not detect any genes of the ATG12 system in piroplasmida, a subgroup of Apicomplexa, and the piroplasm ATG3 does not possess AIM12 associated with the interaction of ATG12. Together, these findings strongly support the evolutionary loss of the ATG12 system in the common ancestor of the piroplasm lineage. 

To predict the presence of AIM8 and AIM12 motifs in ATG3, we conducted AlphaFold-based structural predictions of ATG8-ATG3 and ATG12-ATG3 complexes. Although the presence of AIM8 was predicted in piroplasm ATG3, the amino acids corresponding to AIM12 were missing. In this study, we experimentally demonstrated the binding ability of the predicted AIM8 in *Babesia bovis* to the validity of these predictions. AlphaFold has previously been reported to effectively predict AIM8 [26]. This study demonstrates, for the first time, its effectiveness in predicting AIM8 also in apicomplexan ATG8. Our results highlight the value of combining structural conservation with sequence conservation and predicted protein–protein interactions to enhance functional analyses. This approach is particularly advantageous for studying non-model organisms, where experimental validation of protein functions remains challenging. Additionally, to assess the functionality of ATG8 in piroplasmida, we expressed recombinant BbATG8 and BbATG3 from *B. bovis* and analyzed their interaction. In *P. falciparum*, the interaction between PfATG8 and an AIM8-containing peptide of PfATG3 has been experimentally demonstrated, with their co-crystal structures resolved [16]. In our study, we detected interaction signals between BbATG8 and BbATG3 comparable to the positive control of PfATG8 and PfATG3. And the interaction was dependent on AIM8 in BbATG3. Therefore, the results strongly support that the ATG8 conjugation system is also functional in *B. bovis* through a specific interaction mediated by the AIM8. Together, our results provide the first experimental validation of the molecular mechanism of the ATG8 system in *B. bovis*.

Demonstrating ATG8 conjugation to PE is the most definitive evidence of a functional ATG8 conjugation system (Figure 1). However, we were unable to confirm this for BbATG8 in this study. While *Toxoplasma* ATG8 conjugation to PE has been detected using an overexpression system in HEK cells [5], our attempts to express recombinant BbATG7 in HEK cells were unsuccessful. The difficulty in expressing recombinant *Plasmodium* proteins [27] suggests that similar challenges may apply to its close relative, *Babesia*. For future analyses of the molecular mechanism of autophagy in *B. bovis* and other piroplasms, developing efficient methods for synthesizing functional recombinant ATG proteins will be critical.

What is the physiological significance of losing the ATG12 system? To address this question, it will be essential to clarify the PE conjugation and subcellular localization of ATG8 in piroplasm cells. The ATG12-ATG5-ATG16 complex is known to regulate ATG8 localization [1,2]. In *T. gondii*, this complex is crucial for the apicoplast localization of ATG8 [14], suggesting that ATG8 localization controlled by the ATG12-ATG5-ATG16 complex is a conserved feature in Apicomplexa. Whether ATG8 localizes to the apicoplast in piroplasms remains an open question, as no studies have yet examined ATG8 localization in this lineage. Understanding ATG8 localization in piroplasmida is, therefore, critical not only for elucidating the functional conservation of ATG8 in Apicomplexa but also for comprehensively understanding the biological implications of ATG12 system loss.

For ATGs other than piroplasms, in this study, we found many repetitive sequence insertions in coccidian ATGs. For example, there were conspicuous insertions of repeated sequences of alanine residues in the N-terminal region of ATG3 (Figure 4B) and in the UblB domain of ATG5 (Figure S2) in *Eimeria*. Insertion sequences were also found in ATG8 in *Sarcocystis neurona* and *Cystoisospora suis* (Figure S1). Although it was previously known that *Toxoplasma* ATG12 has a long insertion sequence upstream of the ubiquitin structure [5], we found that this insertion sequence is present in all species of the later lineages that diverged from the genus *Eimeria* (Table S3). An elongation sequence is also present at the N-terminus of ATG12 in other organisms, which is an intrinsically disordered region (IDR) that does not take on a specific structure. Recently, it was reported that the IDR of yeast Atg12 functions to facilitate interactions with other Atg proteins and to protect its own ubiquitin structure [28]. Although it has been reported that in coccidia there are many insertions of specific amino acid repeat sequences [29,30], it is interesting to note that these sequence insertions are also present in a group of ATG proteins that are universally conserved in eukaryotes, considering the functional evolution of the proteins. Importantly, the conspicuous repeat sequences within CDS regions in *Eimeria* are likely to hinder blast searches, which might explain why ATG12 could not be identified in *Eimeria necatrix* and *Cyclospora cayetanensis*. Given the possibility that IDRs may contribute to protein protection, examining the intracellular proteolytic activity (autophagy and proteasomes) of coccidian parasites may provide an explanation for the evolution of the acquisition of their prominent insertions.

Our findings collectively indicate that the ATG12 system was lost in the common ancestor of piroplasms, a subgroup within Apicomplexa. Zhang et al. [7] proposed that the ATG8 and ATG12 conjugation systems originated before the emergence of eukaryotes, with the ATG8 system appearing first, followed by the ATG12 system, which likely arose through gene duplication. If this scenario is accurate, the loss of the ATG12 system in piroplasms may reflect an ancestral state of the ATG8 system predating the evolution of the ATG12 system. Future studies of the molecular mechanisms of ATG8 localization in piroplasms may reveal simplified E3 enzymes from the ATG12–ATG5 conjugate or novel alternative factors. Functional analysis of these factors will reveal the minimal features required for the E3 enzyme to ATG8, which will provide critical insights into the evolutionary trajectory of the ATG12–ATG5 conjugate and its functional principles. 

## Data Availability

The sequences of the identified ATG homologs in this study are attached in the Supplementary Materials.

## Supplementary Materials

The following supporting information can be downloaded at https://www.mdpi.com/article/10.3390/cells14020121/s1: Figure S1: An alignment of ATG8 homologs in Apicomplexa used in this study with model organisms; Figure S2: An alignment of ATG5 homologs in the *Eimeria* genus with model organisms; Figure S3: AlphaFold predicted structure models colored by the model confidence level (related to Figure 3A and Figure 4A); Figure S4: AlphaFold predicted structure models colored by the model confidence level (related to Figure 5); Table S1: The target genes and primer sequences for PCR; Table S2: Plasmids constructed in this study; Table S3. List of ATG homologs for the ATG8 and ATG12 systems in this study.
