# Supplementary material for: Discovery of Evolutionary Loss of the Ubiquitin-like Autophagy-Related ATG12 System in a Lineage of Apicomplexa
Source: Cells. 2025 Jan 15;14(2):121. doi: 10.3390/cells14020121 (PMC11764061; doi:10.3390/cells14020121)
Supplement: Supplementary file 1 [file cells-14-00121-s001.zip › Sup-Figure-ATG12loss-Revise-v6.pdf]

Figure S1

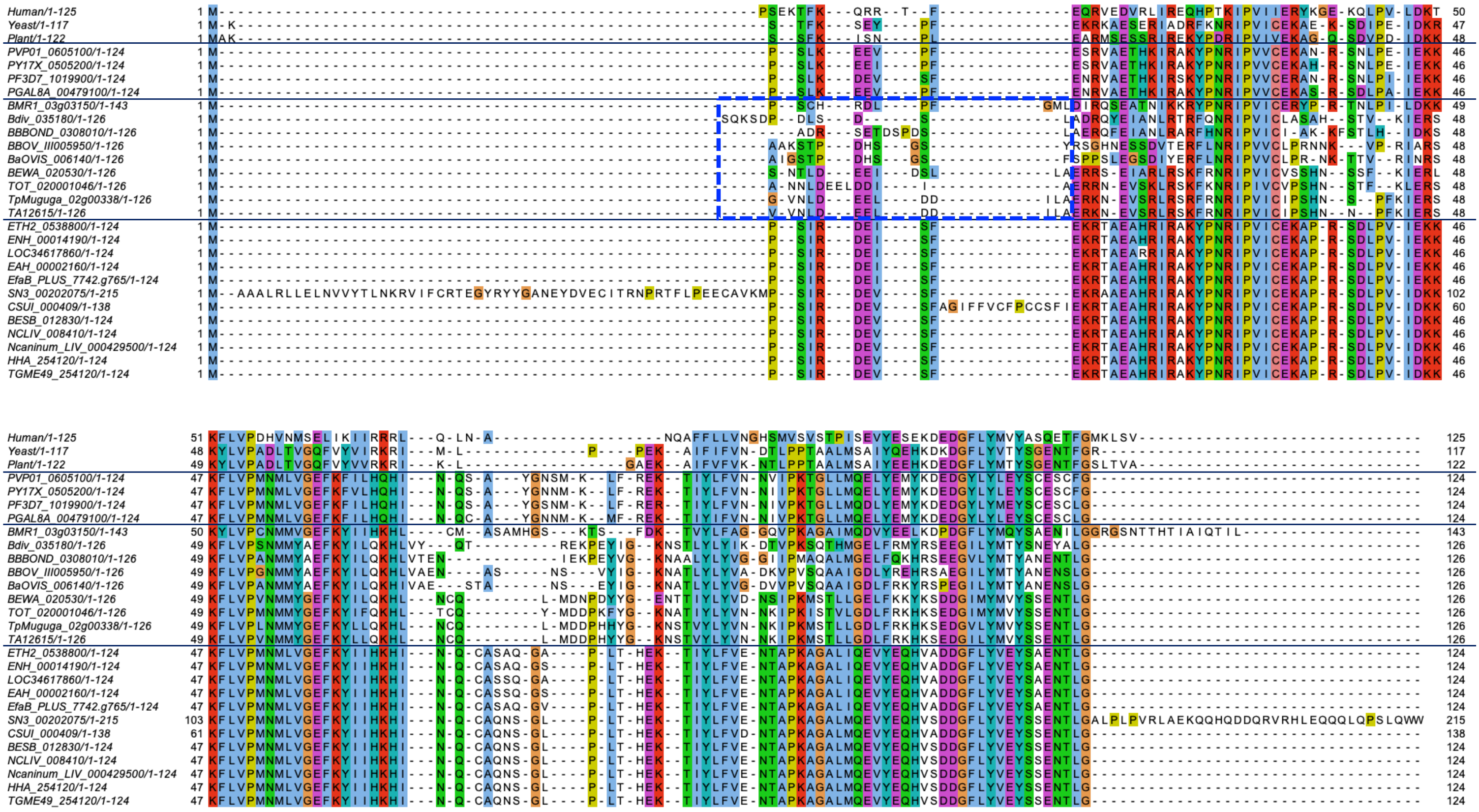

Figure S1. An alignment of ATG8 homologs in Apicomplexa used in this study with model organisms.
The alignment was performed using Expresso (https://tcoffee.crg.eu/apps/tcoffee/do:expresso) and the results vidualised using JalView software. The blue dashed square indicate the N-terminal region of the unique sequence of piroplasm ATG8 homologs.

Figure S2

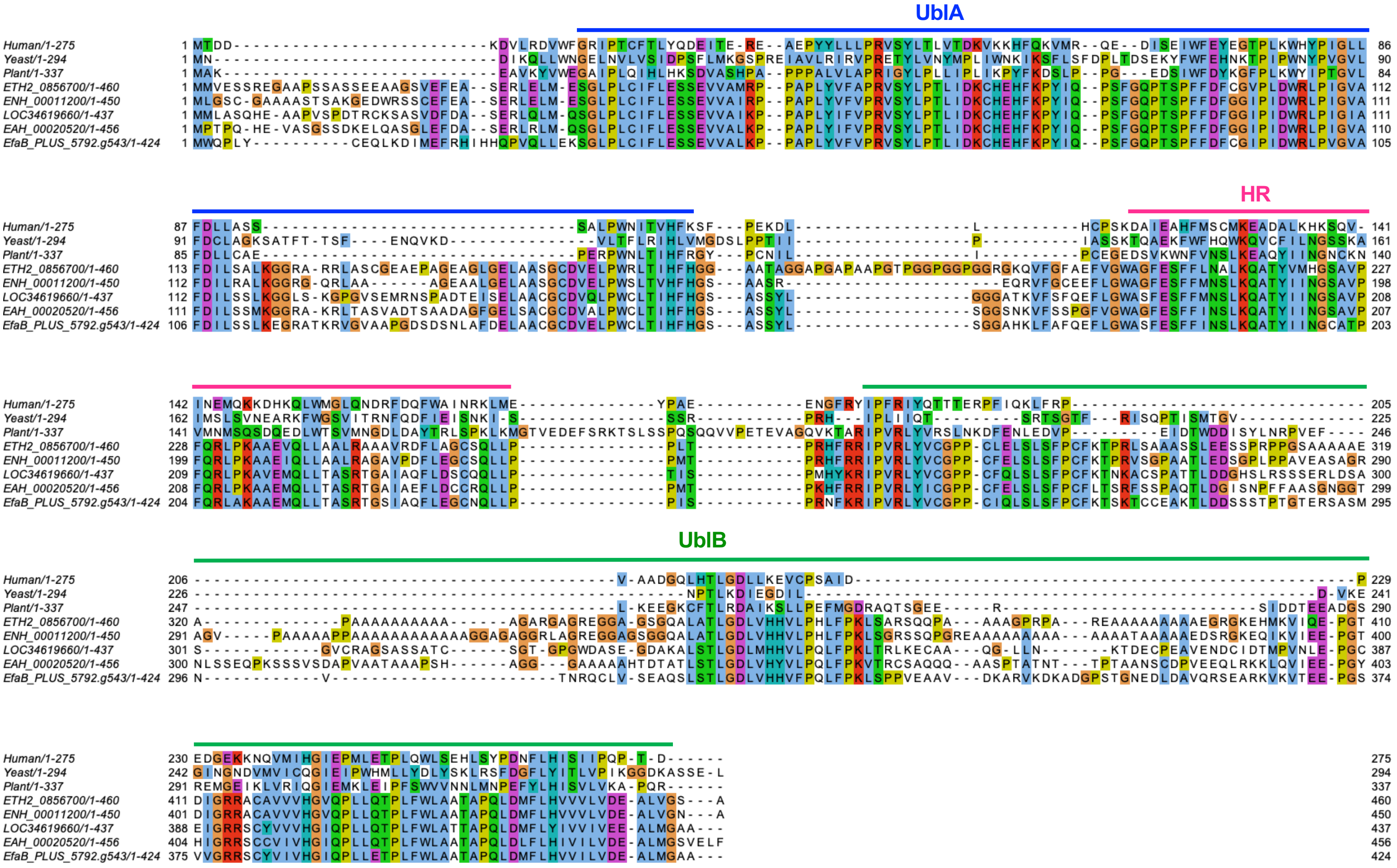

Figure S2. An alignment of ATG5 homologs in *Eimeria* genus with model organisms.
The alignment was performed using Expresso (https://tcoffee.crg.eu/apps/tcoffee/do:expresso) and the results vidualised using JalView software. Two ubiquitin-like domains, UblA and UblB, and helix-rich domain (HR) are shown by the blue, green, and magenta lines, respectively.

Figure S3

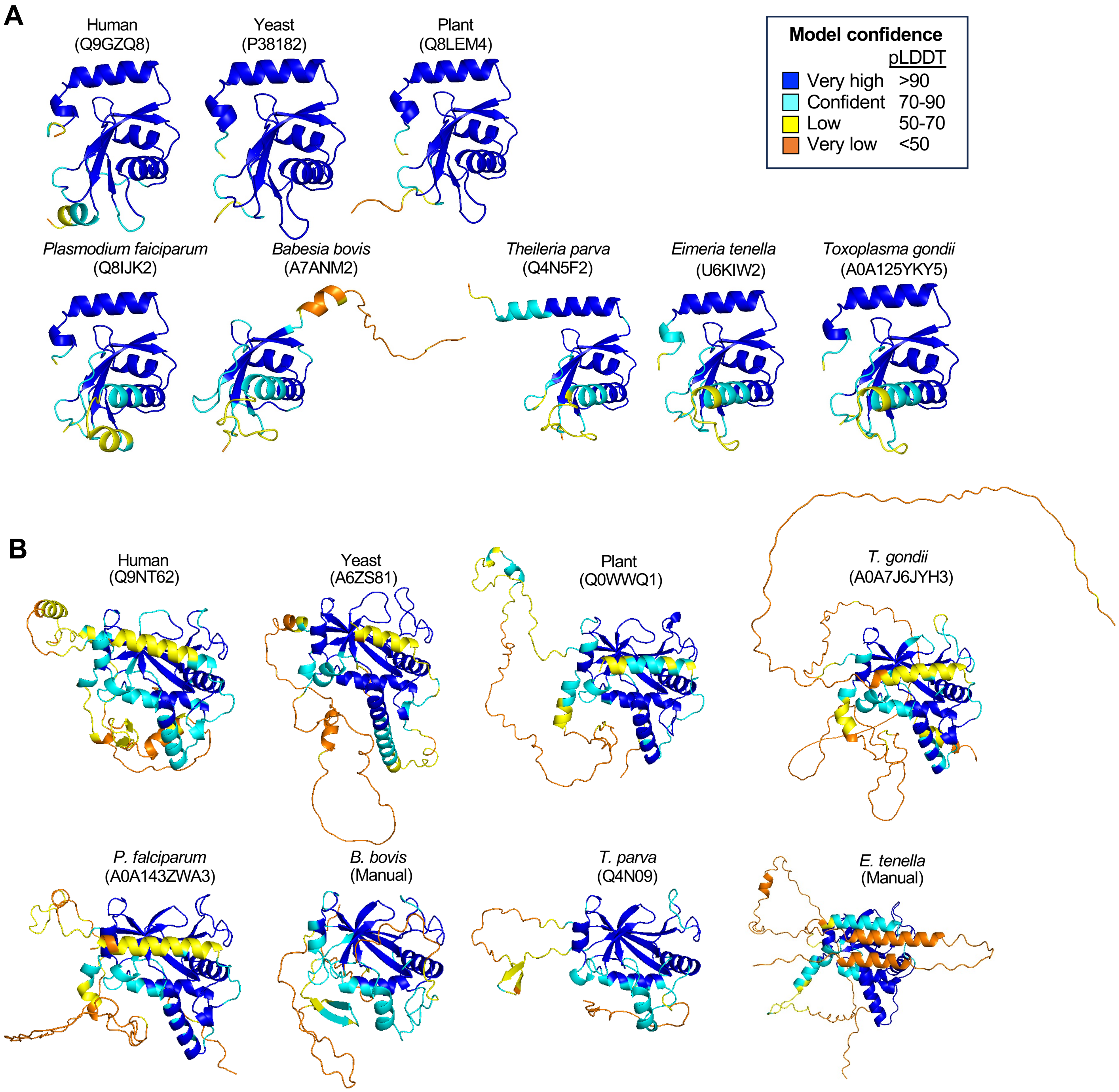

Figure S3. AlphaFold predicted structure models colored by the model confidence level (related to Figure 3A and 4A). AlphaFold 3D structure prediction models using full-length sequences of ATG8 **(A)** and ATG3 **(B)** colored according to the predicted local distance difference test (pLDDT). UniProt number which can be used for searching in AlphaFold Protein Structure Database (https://alphafold.ebi.ac.uk/) are shown. Human: *Homo sapiens*, Yeast: *Saccharomyces cerevisiae*, plant: *Arabidopsis thaliana*.

Figure S4

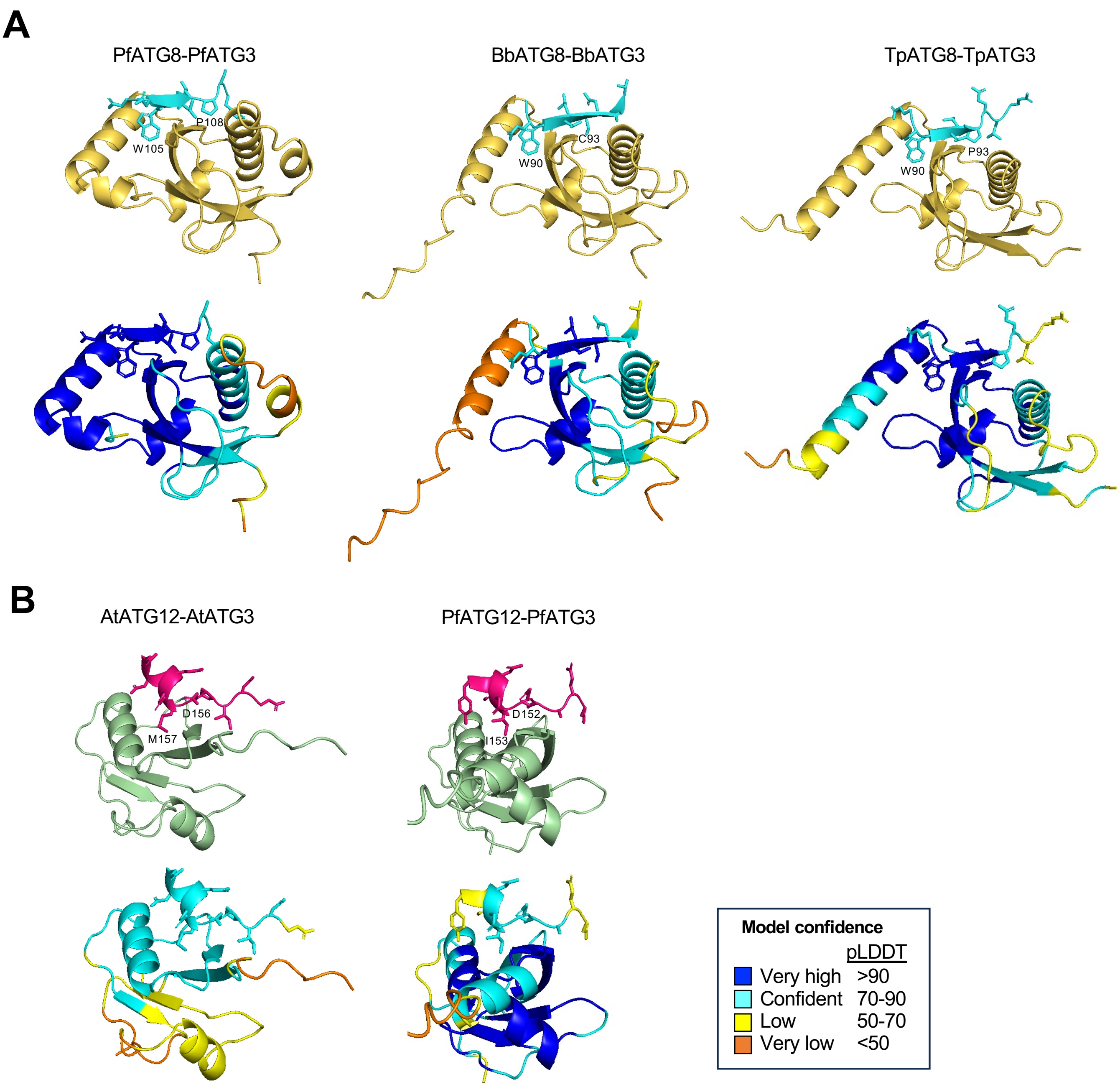

Figure S4. AlphaFold predicted structure models colored by the model confidence level (related to Figure 5). AlphaFold 3D structure prediction models of AIM8 **(A)** and AIM12 **(B)**. Upper: colored by proteins (identical to Figure 5), lower: colored according to the predicted local distance difference test (pLDDT).
